# Immunogenic Comparison of Chimeric Adenovirus 5/35 Vector Carrying Optimized Human Immunodeficiency Virus Clade C Genes and Various Promoters

**DOI:** 10.1371/journal.pone.0030302

**Published:** 2012-01-19

**Authors:** Masaki Shoji, Shinji Yoshizaki, Hiroyuki Mizuguchi, Kenji Okuda, Masaru Shimada

**Affiliations:** 1 Department of Molecular Biodefense Research, Yokohama City University, Yokohama, Japan; 2 Laboratory of Biochemistry and Molecular Biology, Graduate School of Pharmaceutical Sciences, Osaka University, Osaka, Japan; 3 Laboratory of Gene Transfer and Regulation, National Institute of Biomedical Innovation, Ibaraki, Osaka, Japan; 4 Choju Medical Institute, Fukushimura Hospital, Toyohasi, Aichi, Japan; University of Pittsburgh Center for Vaccine Research, United States of America

## Abstract

Adenovirus vector-based vaccine is a promising approach to protect HIV infection. However, a recent phase IIb clinical trial using the vector did not show its protective efficacy against HIV infection. To improve the vaccine, we explored the transgene protein expression and its immunogenicity using optimized codon usage, promoters and adaptors. We compared protein expression and immunogenicity of adenovirus vector vaccines carrying native or codon usage-optimized HIV-1 clade C gag and env genes expression cassettes driven by different promoters (CMV, CMVi, and CA promoters) and adapters (IRES and F2A). The adenovirus vector vaccine containing optimized gag gene produced higher Gag protein expression and induced higher immune responses than the vector containing native gag gene in mice. Furthermore, CA promoter generated higher transgene expression and elicited higher immune responses than other two popularly used promoters (CMV and CMVi). The second gene expression using F2A adaptor resulted in higher protein expression and immunity than that of using IRES and direct fusion protein. Taken together, the adenovirus vector containing the expression cassette with CA promoter, optimized HIV-1 clade C gene and an F2A adaptor produced the best protein expression and elicited the highest transgene-specific immune responses. This finding would be promising for vaccine design and gene therapy.

## Introduction

The human immunodeficiency virus (HIV), responsible for acquired immune deficiency syndrome (AIDS), is classified into type 1 and type 2. HIV type 1 (HIV-1) is further classified into 11 phylogenetically related genetic subtypes (clades) from A to K. HIV-1 clade B is dominant in developed countries, such as North America and Western Europe, and has been the primary focus of vaccine development. A phase IIb clinical trial of an adenovirus serotype 5 (Ad5) vector-based HIV-1 IIIb vaccine (STEP trial) failed to protect against HIV infection [1], [2]. Further, HIV-1 clade C is the most dominant subtype (approximately half of HIV infected people) [3] in the world, especially in developing countries such as India [4], China [5], and the Sub-Saharan African countries [6]. Therefore, the development of a vaccine against HIV-1 clade C is urgently required.

In this study, we investigated the immunogenicity of HIV clade C genes driven by three promoters and with two adaptor sequences in a bicistronic Ad vector. We used the replication-defective Adenovirus type 5 (Ad5) vector containing adenovirus type 35 fiber (Ad5/35) as a vaccine vector [7]. Ad5/35, in common with Ad35, uses CD46 as a receptor for infection [8]. CD46 is expressed on the majority of human cells, enabling the Ad5/35 vector to transduce a wide range of human tissues. The tropism to the liver and hepatotoxicity of Ad5/35 was much lower than for Ad5 [9].

The immunogenicity of a vaccine vector is not only dependent on the vector but also on the magnitude of antigen expression. Several promoters have been widely studied in mammalian cells and animals, such as virus-origin promoters (CMV, CMVi, CA, Sra, and LTR promoters) and tissue-origin promoters (EF1a and p53 promoters). The CMV promoter has been widely used for expression in mammalian cells, since it is much strong compared to other promotes. To optimize an Ad vector-based HIV clade C vaccine, we compared the expression and immunogenicity of HIV-1 clade C genes driven by different promoters (CMV, CMVi, and CA promoters) and adapters (IRES and F2A), using native and optimized genes. It has been reported that optimized genes showed higher protein expression *in vitro* and the immunogenicity of a DNA vaccine *in vivo* was higher when compared to native genes [10], [11]. The CMV promoter is derived from the human cytomegalovirus immediate-early 1 gene promoter, and the CMVi promoter consists of the CMV promoter and an intron A. The CA promoter consists of the CMV enhancer and the chicken β-actin promoter with the chicken β-actin intron. Plasmids with the CMVi promoter or the CA promoter have higher levels of gene expression than plasmids with the CMV promoter, both *in vitro* and *in vivo* [12]. To express the Gag and Env proteins in a bicistronic vector, we used internal ribosome entry site (IRES) sequences [13], [14], furin cleavage site and 2A (F2A) sequences, or fusion types of gagopt and envopt. IRES sequences derived from the encephalomyocarditis virus (EMCV) are commonly used in gene therapy and gene transfer experiments [15], but the expression of the IRES-dependent second gene is much lower than the expression of the catabolite activator protein (cap)-dependent first gene [16]. The F2A adaptor is linked to a furin cleavage site in the foot-and-mouth-disease virus (FMDV) 2A sequence [17], [18]. F2A has been shown to express high levels of a full-length, functional monoclonal antibody (mAb), *in vitro* and *in vivo*, where F2A was used to link the heavy and light chains of the mAb [17], [18].

In this study, we explored the immunogenicity of adenovirus vectors with different promoters, adaptors, and genes. The best transgene expression and immunogenicity were obtained from an adenovirus vector containing the CA promoter with an F2A adaptor and an optimized transgene.

## Results

### *In vitro* protein expression analysis of the HIV-1 clade C gag gene

**Figure 1:**
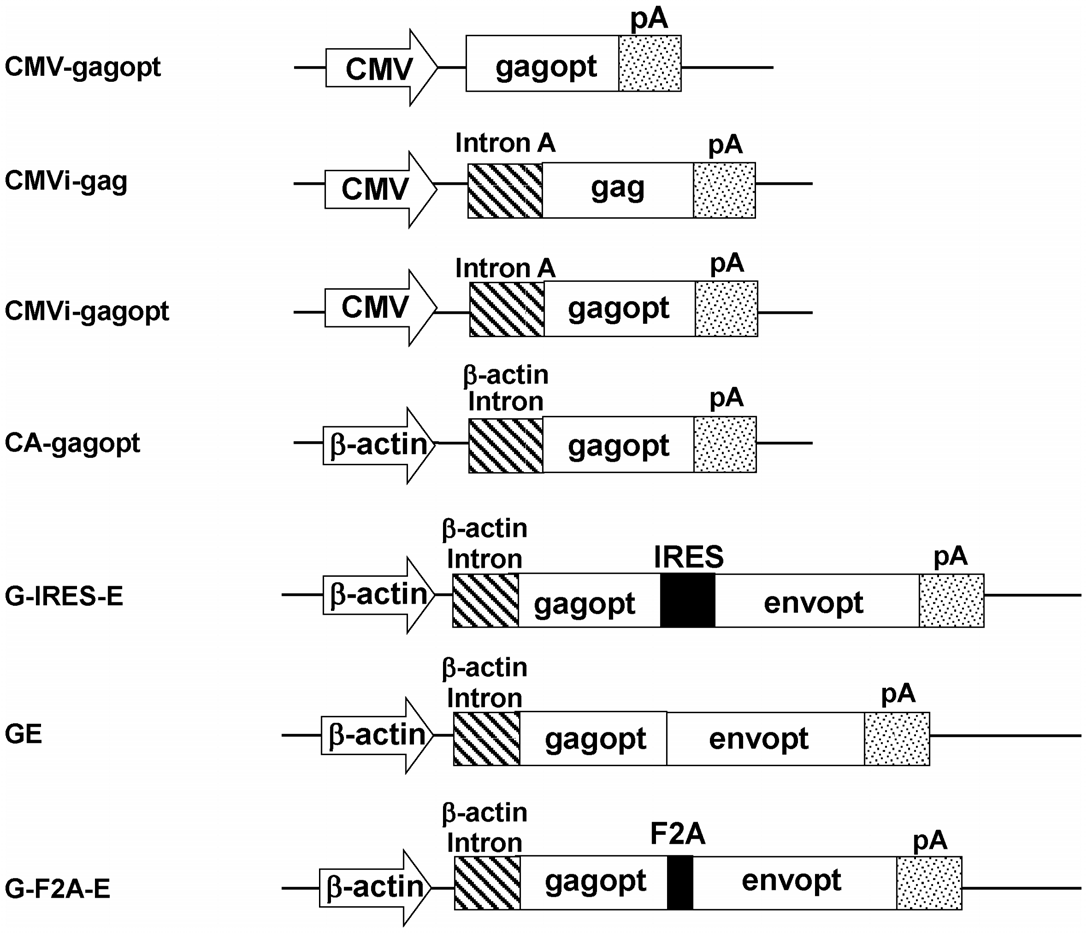
Schematic design of the expression cassettes used in this study. CMV, CMV immediate promoter and enhancer (CMV-IE); CMVi, CMV-IE with intron A; CA, CMV enhancer and chicken β-actin promoter with β-actin intron; gagopt, optimized HIV_96ZM651_ gag coding gene; gag, native HIV_96ZM651_ gag coding gene; envopt, optimized HIV_96ZM651_ envelope gp160 coding gene; IRES, internal ribosome entry site sequence from the encephalomyocarditis virus; F2A, a combination of furin cleavage site and 2A self-processing sequence; pA, polyadenylation signal sequence.

**Figure 2:**
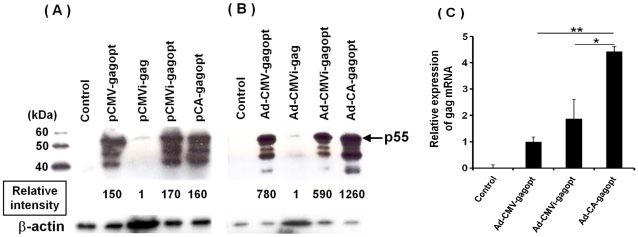
Analysis of HIV clade C Gag protein expression. HeLa cells were transfected with 2 µg of plasmid (pCMV-gagopt, pCMVi-gag, pCMVi-gagopt, or pCA-gagopt) (A) or infected with the corresponding Ad vector at MOI 5 (Ad-CMV-gagopt, Ad-CMVi-gag, Ad-CMVi-gagopt, or Ad-CA-gagopt) (B). Untreated HeLa cells were used as a negative control. After two days of incubation, the cells were harvested, and proteins were separated by sodium dodecyl sulfate-polyacrylamide gel electrophoresis (SDS-PAGE) electrophoresis, transferred, and assayed by western immunoblotting using a Gag-specific Ab. To calculate the Gag protein relative intensity, the cell lysates from pCMVi-gag or Ad-CMVi-gag constructs were 10- or 50-fold more loaded in these lanes than in any of the other lanes, respectively. The number indicates the mean relative protein intensity to the Gag protein. Data are representative of five independent experiments. (C) Analysis of gagopt mRNA expression in HeLa cells infected with Ad-CMV-gagopt, Ad-CMVi-gagopt or Ad-CA-gagopt by quantitative real-time PCR. HeLa cells were infected with the corresponding Ad vector at MOI 2 (Ad-CMV-gagopt, Ad-CMVi-gagopt or Ad-CA-gagopt). After two days of incubation, total RNA was isolated from infected cells and cDNA was generated. cDNA was used as a template for quantitative real-time PCR. Data represent the mean ± standard error of the mean (S.E.M.) and are representative of two independent experiments. *, *p*<0.05; **, *p*<0.01.

To compare the expression efficiency of the HIV clade C Gag protein among several gag expression cassettes (Figure 1) *in vitro*, HeLa cells were transduced with plasmids (pCMV-gagopt, pCMVi-gag, pCMVi-gagopt, or pCA-gagopt) or infected with Ad vectors (Ad-CMV-gagopt, Ad-CMVi-gag, Ad-CMVi-gagopt, or Ad-CA-gagopt) (Figure 1). After two days of incubation, Gag protein expression was analyzed by western blotting with an anti-HIV clade C gag p24 mAb, and the intensity of Gag was quantified with Image J software. p55^Gag^ and two or three extra proteins containing p24 were detected (Figures 2A and 2B). We considered that these extra proteins were incompletely processed proteins with cellular proteases and speculate as follows: the 48 kDa band may consist of p17, p24 and p7; the 42 kDa band may consist of p17 and p24; and the 38 kDa band may consist of p24, p7 and p6. We calculated the relative protein intensity of total Gag protein expression to the levels of the native Gag protein. In the context of the influence of the gag gene sequence, the optimization of the gene sequence increased its expression to approximately 170 fold in plasmid-transfected cells (pCMVi-gagopt *vs* pCMVi-gag), and 590 fold in Ad-infected cells (Ad-CMVi-gagopt *vs* Ad-CMVi-gag) (Figures 2A and 2B). No remarkable difference of Gag protein expression was observed in the cells transfected with gagopt-expressing plasmids with different promoter (CMV, CMVi and CA) (Figure 2A). However, CA promoter driving vector (Ad-CA-gagpot) expressed two-fold higher Gag protein than the vectors driven by CMV promoter (Ad-CMV-gagopt) and CMVi promoter (Ad-CMVi-gagpot). Significant higher gag mRNA in HeLa cells infected with Ad-CA-gagopt vector was observed than other two vectors (Ad-CMV-gagopt and Ad-CMVi-gagopt, *p*<0.01 and *p*<0.05, respectively) (Figure 2C). Taken together, we found that gagopt expression cassette driven by CA promoter is the best expression efficiency of the HIV clade C Gag protein in our cassettes indicated in figure 1.

### Immunogenicity of the clade C gag expression cassette in the Ad vectors

**Figure 3:**
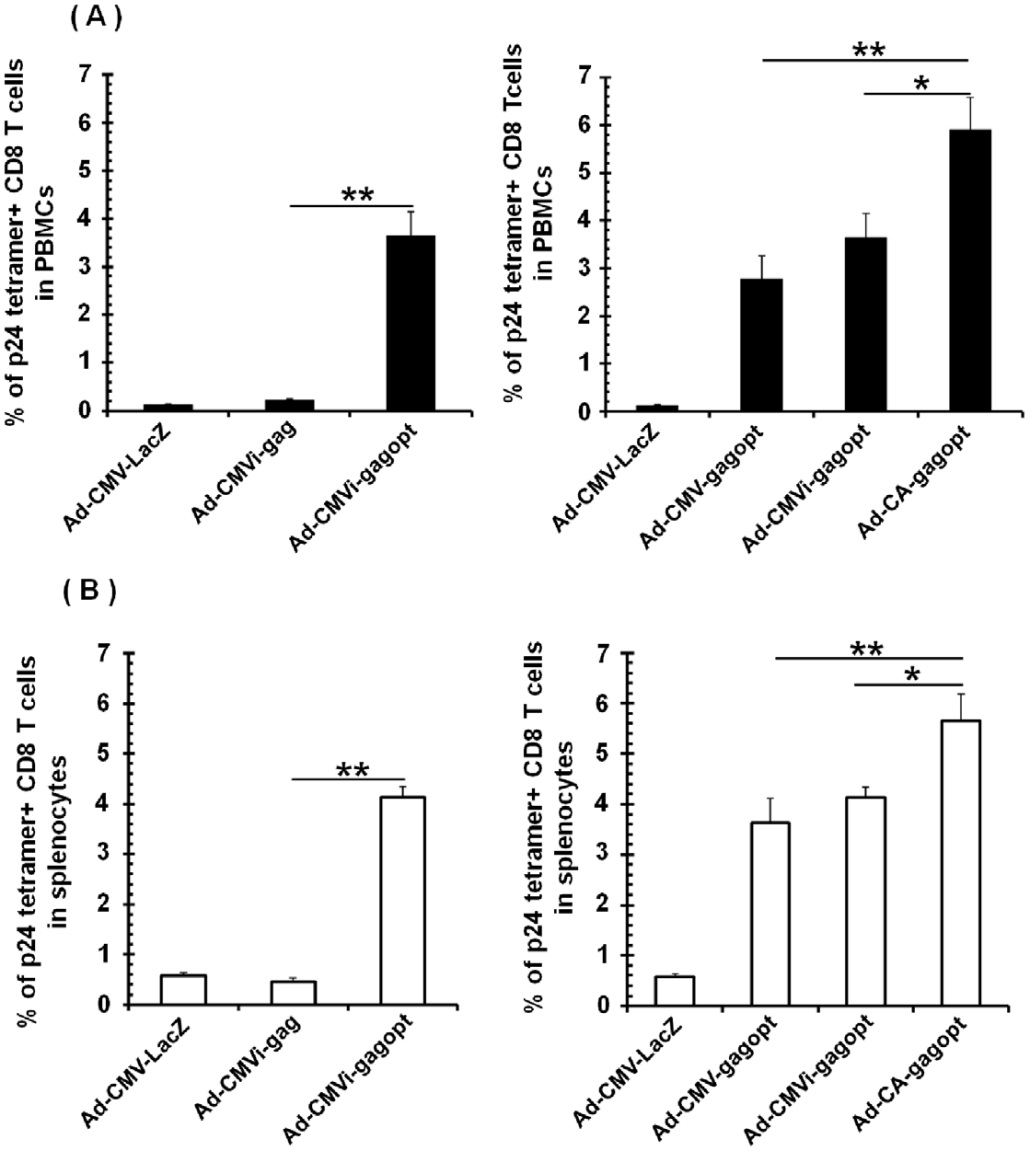
HIV Gag-specific CD8 T cells in mice. Balb/c mice (5–6/group) were immunized with 1×10^8^ pfu of Ad vector (Ad-CMV-gagopt, Ad-CMVi-gag, Ad-CMVi-gagopt, Ad-CA-gagopt, or Ad-CMV-LacZ) with intramuscular injection. Ad-CMV-LacZ was used as a negative control vector. Ten days post-immunization, the frequency of clade C Gag-specific CD8 T cells in PBMCs (A) or splenocytes (B) was measured by the H-2Dd/p24 tetramer assay in 5-6 individual mice. Data represent the means ± S.E.M. and are representative of two independent experiments. *, *p*<0.05; **, *p*<0.01.

To investigate the immunogenicity of the Ad vectors, BALB/c mice were intramuscularly immunized with Ad-CMV-gagopt, Ad-CMVi-gag, Ad-CMVi-gagopt, Ad-CA-gagopt, or Ad-CMV-LacZ (Figure 1). Ten days post-immunization, the frequency of Gag-specific CD8 T cells in PBMCs (Figure 3A) and splenocytes (Figure 3B) was measured with H-2D^d^/p24 tetramer (AMQMLKDTI). The tendency of the immune response against Gag in PBMCs was similar with that in splenocytes. A strong Gag-specific cell-mediated response was detected in the mice immunized with gagopt-expressing Ad vector (Ad-CMVi-gagopt), but not with naïve gag-expressing Ad vector (Ad-CMVi-gag) (Figures 3A and 3B), demonstrating that corresponding to the results of mRNA and protein expression, optimized gene is better than native viral gene for virus-based vaccine vector.

Then we investigated the immunogenicity of Ad vectors expressing optimized gag gene driven by various promoters. Again, The CA promoter driven Ad vector (Ad-CA-gagopt) induced significantly higher Gag-specific CD8 T cells than the vector driven by CMVi promoter (Ad-CMVi-gagopt) and CMV promoter (Ad-CMV-gagopt) (*p*<0.05) (Figures 3A and 3B), demonstrating that CA promoter elicits higher protein expression and immune responses in viral vector than other two promoters.

**Figure 4:**
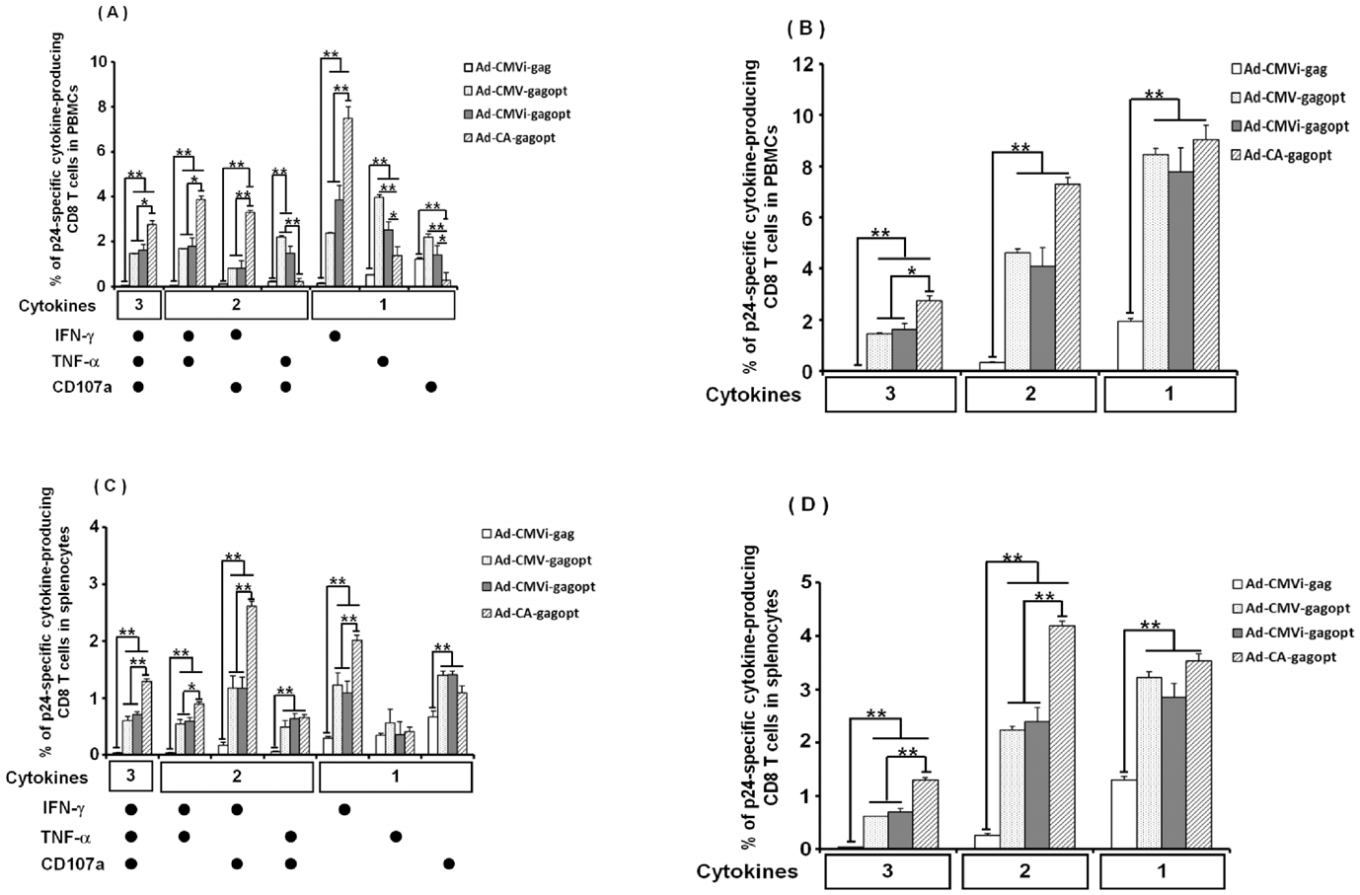
Polyfunctional analysis of HIV clade C Gag-specific CD8 T cells. The same murine samples shown in Fig. 3 were tested by multicolor ICS assay for production of INF-γ, TNF-α and CD107a. (A) or (C) are shown for each individual combination of functions of HIV clade C Gag-specific CD8 T cellsin PBMCs or splenocytes, respectively. (B) or (D) are shown for each combination of functions (three, INF-γ+TNF-α+CD107a+; two, INF-γ+TNF-α+,INF-γ+CD107a+ and TNF-α+ CD107a+; one, INF-γ+, TNF-α+and CD107a+) of HIV clade C Gag-specific CD8 T cells in PBMCs or splenocytes, respectively. These were measured by multicolor ICS assay following stimulation with clade C p24 peptide. IFN-γ, TNF-α and CD107a were used as the functional markers. This assay was performed in 5-6 individual mice. Data represent the means ± S.E.M. and are representative of two independent experiments. *, *p*<0.05; **, *p*<0.01.

Recently, it has been reported that polyfunctional CD8 and CD4 T cell responses, which produce multiple cytokines in a single-cell, are associated with lower plasma viral loads in patients with chronic HIV infection [19], [20]. Therefore, we evaluated the HIV-1 clade C Gag-specific cell-mediated immune response by multicolor intracellular cytokine staining (ICS) and the productivity of INF-γ, TNF-α and CD107a in PBMCs and splenocytes ten days post-immunization. As shown in Figure 4, gagopt-expressing Ad vectors produced significantly higher cytokine-producing CD8 T cells (including single, double and triple cytokine-secreting cells) in both PBMCs (Figure 4A, 4B) and splenocytes (Figure 4C, 4D) than the naïve gag-expressing Ad vector (Ad-CMVi-gag). CA promoter driven Ad vector (Ad-CA-gagopt) produced further higher polyfuctional cytokine-producing CD8 T cells than other promoter-driven Ad vectors. These results demonstrate that Ad-CA-gagopt induces higher polyfunctional HIV-1 clade C Gag-specific CD8 T cells than that of Ad-CMVi-gag, Ad-CMV-gagopt or Ad-CMVi-gagopt in BALB/c mice.

**Figure 5:**
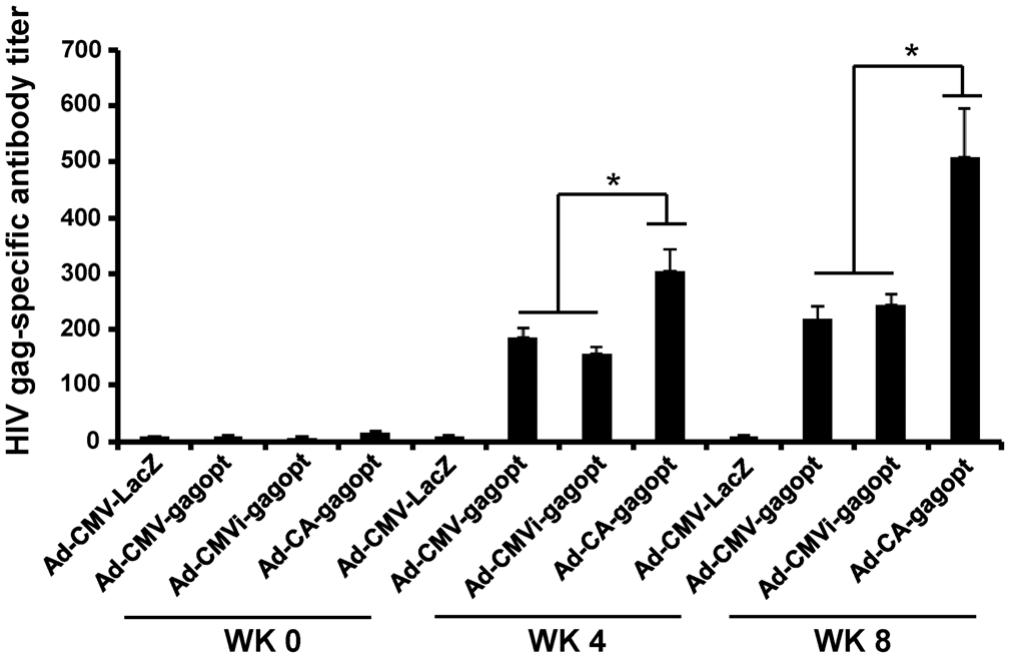
HIV clade C Gag-specific humoral immune response. Eight weeks post-immunization, HIV clade C Gag-specific Ab titer was measured by ELISA. Diluted immune sera from 800 to 12,800-fold were used for ELISA. The ELISA plate was coated with HIV clade C p24 protein. The detection of Gag-specific Ab titer was performed at an absorbance of 450 nm. This assay was performed in 5-6 individual mice. Data represent the means ± S.E.M. and are representative of two independent experiments. *, *p*<0.05.

To investigate HIV-1 clade C Gag-specific humoral immune responses, antibody (Ab) against Gag was measured 8 weeks after immunization by ELISA (Figure 5). As we observed with the cell-mediated responses, the Ad-CA-gagopt induced significantly higher gag-specific Ab (*p*<0.05) than the other vaccines. Similar level of Ab was induced by Ad-CMV-gagopt and Ad-CMVi-gagopt. These results indicate that Ad-CA-gagopt induces higher HIV-1 clade C Gag-specific humoral immune responses than that of Ad-CMVi-gag, Ad-CMV-gagopt or Ad-CMVi-gagopt in BALB/c mice.

In conclusion, we found that immunization of Ad-CA-gagopt to mice induced higher Gag-specific polyfunctional Gag-specific cell-mediated immunity and humoral immunity than that of Ad-CMVi-gag, Ad-CMV-gagopt or Ad-CMVi-gagopt.

### Protein expression and immunogenicity of the HIV-1 clade C env gene downstream of the gag gene

**Figure 6:**
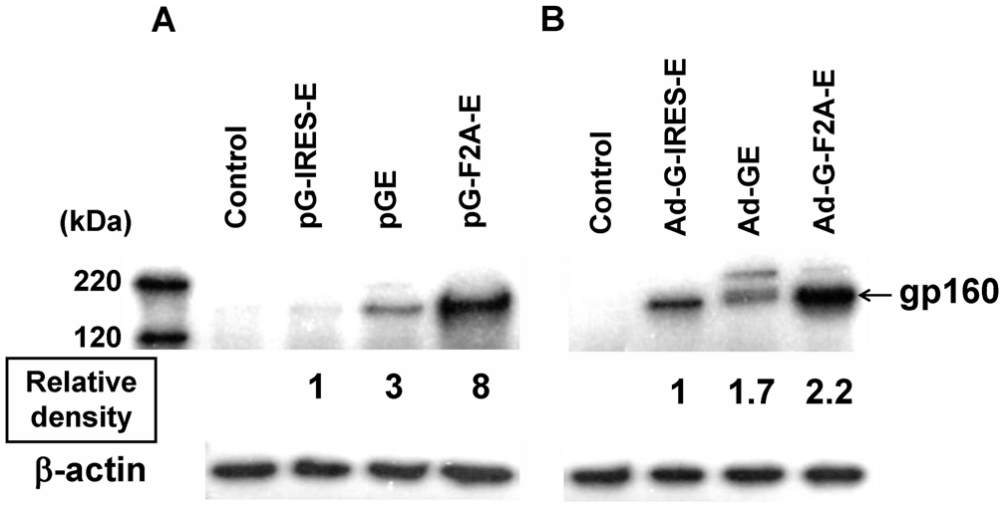
Analysis of the expression of HIV clade C Env protein from the bicistronic vectors. HeLa cells were transfected with plasmid (pG-IRES-E, pGE, or pG-F2A-E) (A) or infected with Ad vector (Ad-G-IRES-E, Ad-GE, or Ad-G-F2A-E) at MOI 5 (B) (Fig. 1). Untreated HeLa cells were used as a negative control. After two days of incubation, the cells were harvested and proteins were separated by SDS-PAGE electrophoresis, transferred and assayed by western immunoblot using an Env-specific Ab (HIV clade C gp120 mAb). The number indicates the mean relative protein intensity to the expression of the Env protein containing the IRES cassette. Data are representative of five independent experiments.

To investigate the expression efficiency of the second gene, env was introduced to downstream of gag in a bicistronic vector. We constructed vectors containing a fusion type protein without any adapter sequences (GE) or with the adapter sequences, IRES (G-IRES-E) or F2A (G-F2A-E). These expression cassettes were separately introduced to a plasmid or the Ad vector. HeLa cells were then transduced with these bicistronic vectors. After 2 days of incubation, Env protein expression was analyzed by western blotting with an anti-HIV clade C Env Ab (Figure 6). We used β-actin protein, not Gag protein, as an internal control, because Gag protein was expressed as a fusion protein with Env by plasmid pGE or adenovirus vector Ad-GE. On membrane probed with the anti-Env Ab, the upper band was estimated to be the fusion protein of Gag and Env, and the lower band as Env protein including the fusion protein that was generated by cellular proteases. The intensity of the Env protein was analyzed with Image J software and the relative intensity from pG-IRES-E (Figure 6A) or Ad-G-IRES-E (Figure 6B) were shown. In both cases, the env gene that was linked to the gag gene by the F2A sequence was expressed most effectively (Figures 6A and 6B). The Gag protein expression in these bicistronic vectors was equivalence (data not shown). Taken together, we found that the env expression efficiency introduced downstream of gag in a bicistronic vector with F2A is the best expression efficiency of HIV-1 clade C Env protein in our cassettes indicated figure 1.

To explore the peptide for ICS of HIV clade C env, we i.m. immunized mice with 10^8^ pfu of Ad-G-F2A-E. Ten days after immunization, mouse splenocytes were isolated and stimulated with HIV consensus clade C env pool (obtained from AIDS Research and Reference Reagent Program). The peptide pool contains 212 peptide with 15-mer amino acid and 10-mer overlap each other. We separated the peptides to 12 groups (group1-10 contains 20 peptides per group, group 11 contains 12 peptides, and one group contains all 212 peptides). Env-specific IFN-γ-secreting CD8 T cells were detected from mouse splenocytes. We found 0.313%, and 0.116% of env-specific response was observed in the splenocytes stimulated with group 10 (env region 721-811 amino acid), and all pool (env region 1-856 amino acid), respectively (data not shown). Other peptide groups induced 0.016%–0.082% IFN-γ-secreting CD8 T cells. Then we used group 10 peptide pool to detect HIV clade C env-specific cell-mediated responses.

**Figure 7:**
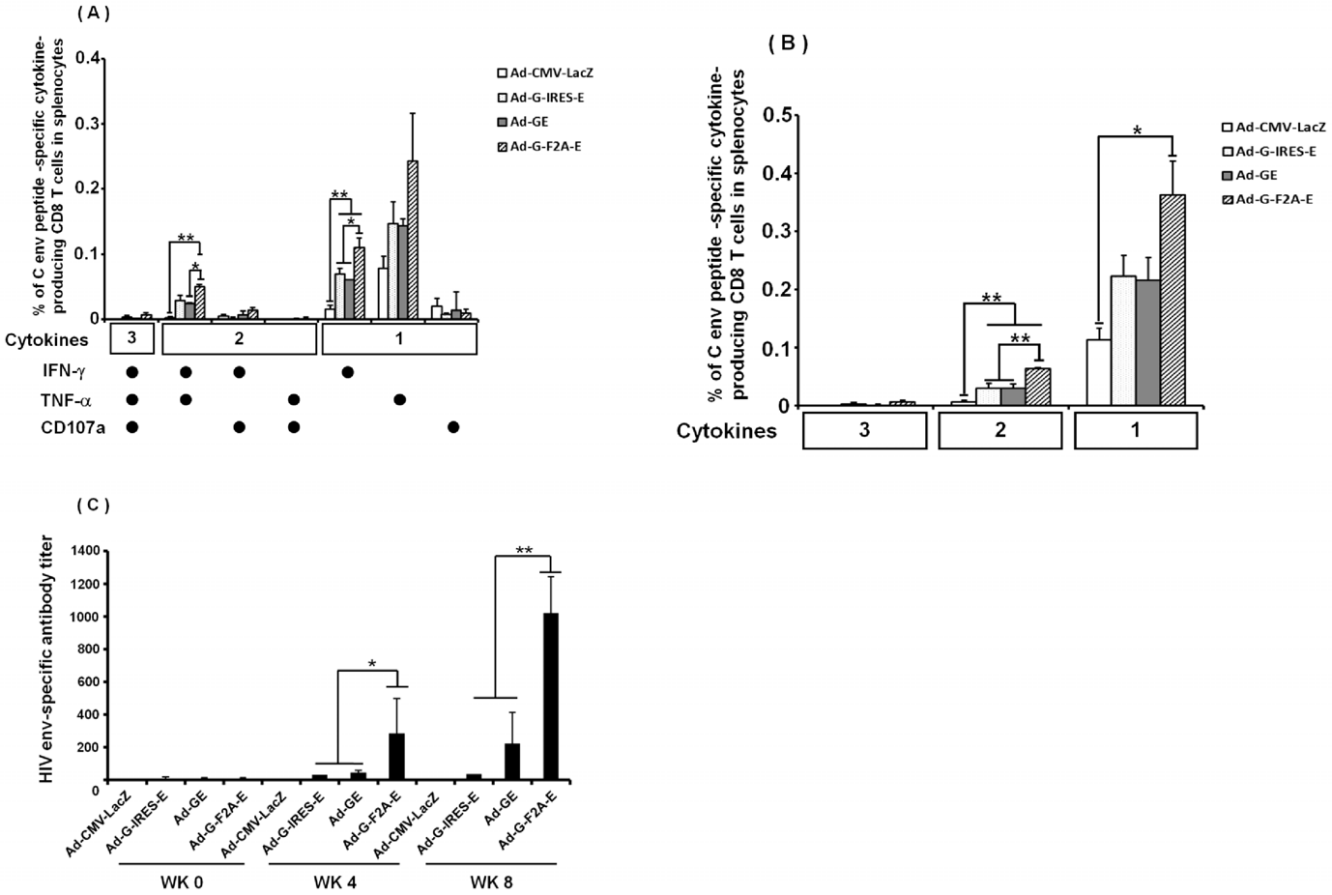
HIV clade C Env-specific humoral immune response. HIV clade C Env-specific Ab titer was measured by ELISA. The mice (6/group) were immunized with 1×10^8^ pfu of Ad vector (Ad-G-IRES-E, Ad-GE, or Ad-G-F2A-E). Eight hundred-fold diluted immune sera were used for ELISA. The ELISA plate was coated with HIV clade C Env peptide. The detection of Env-specific Ab titer was performed at an absorbance of 450nm. This assay was performed with 6 individual mice sera. Data represent the means ± S.E.M. and are representative of two independent experiments. *, *p*<0.05; **, *p*<0.01.

To explore the immunogenicity of the second gene, we immunized mice with Ad-G-IRES-E, Ad-GE, or Ad-G-F2A-E. Ten days after immunization, mouse splenocytes were isolated and stimulated with the group 10 peptide pool. As shown in figures 7A and 7B, significantly higher IFN-γ-single- and IFN-γ/TNFα-double positive CD8 T cells were observed in vaccine groups than that in control group. Ad-G-F2A-E vector induced higher IFN-γ-single- and IFN-γ/TNFα-double cytokine producing CD8 T cells than other vaccines. Furthermore, Ad-G-F2A-E also induced significantly higher levels of Env-specific Ab at 4, 6 and 8 weeks after the immunization as compared to the other vaccines (Figure 7C, *p*<0.05 at 4 and 6 weeks, *p*<0.01 at 8 weeks). In conclusion, we found that immunization of Ad-G-F2A-E to mice induced higher env-specific cellular and humoral immunity than that of Ad-G-IRES-E, Ad-GE.

## Discussion

To optimize an HIV clade C vaccine based on the Ad vector, we compared the immunogenicity of HIV clade C genes driven by various elements in mice. As expected, gagopt induced stronger immunity than native gag. Moreover, we compared the immunogenicity of gagopt controlled by three different promoters, the CMV, CMVi, and CA promoters. The Ad vector with the CA promoter induced the most Gag-specific CD8 T cells, polyfunctional CD8 T cells and Gag-specific Ab. Among the bicistronic vectors, the F2A-dependant env gene induced more Env-specific Ab titers.

It has been reported that plasmid-introduced optimized gag induced higher immunity [10] but Ad-based vaccines with optimized genes have not been studied. We observed that gagopt was expressed more efficiently in HeLa cells as compared to native gag. This was probably because the human optimized gag gene was translated easier than native gag.

HIV-specific cytotoxic T lymphocytes (CTL) are important for the suppression of HIV replication, because vaccine-induced virus-specific CTL responses emerge during HIV acute infection and reach to the peak at 7 to 14 days [21], [22], [23], [24]. It has been reported that the HIV core protein, Gag-specific T cell reaction affects the suppression of HIV replication in HIV or Simian Immunodeficiency Virus (SIV) carriers [25], [26], [27]. In this study, mice were intramuscularly immunized with an Ad vector expressing HIV clade C gag, and ten days post-immunization that are the peak of Gag-specific CTL responses, the immunogenicity was analyzed with the p24 tetramer assay and multicolor ICS. HIV elite controllers possess HIV-specific polyfunctional CD8 T cells, and it is important to analyze CD8 T cells polyfunctionally and not monofunctionally [19], [28]. We analyzed mouse CD8 T cells with antibodies recognizing INF-γ, TNF-α or CD107a [29] as T cell functional markers. Ad-CMVi-gagopt induced stronger immunity than Ad-CMVi-gag. This result correlated with the expression efficiency of gag analyzed by western blot analysis. The comparison between the three types of promoters used in this study demonstrated that the CA promoter induced HIV-specific and polyfunctional cell-mediated immunity more effectively overall but less than for the CMV and CMVi promoters in TNF-α or CD107a single producers (Fig. 4). It is possible that the single producers, except for INF-γ decreased for other functional cell groups were increased in Ad-CA-gagopt immunized mice. On the other hand, such phenomena were not observed in splenocytes, and Ad-CA-gagopt was the most effective at inducing triple producers and double producers, INF-γ and CD107a. There appears to be an inconsistence between data of Fig. 3 and Fig. 4. This is thought to be the one by difference of the character of two assays. Tetramer assay measure antigen-specific T cell numbers, whereas multicolor ICS assay measure antigen-specific T cell functions. These assays are not necessarily equivalent [30], [31].

To express two antigens in a bicistronic vector, we used three different approaches, IRES-, F2A-dependent or a fusion type. The F2A-dependant second gene showed a greater efficiency of expression in HeLa cells and higher immunogenicity in mice. Difficulties regarding the expression efficiency of the second gene have been reported in the use of IRES elements and fusion type genes. IRES-dependant expression can be significantly lower than cap-dependant expression [16]. On the other hand, F2A-dependant expression was very stable in cell lines and in mice [32]. We speculate that this difference in expression efficiency of the second gene depends on the mode of expression. IRES sequences allow the initiation of translation in a cap-independent way [15] and F2A sequences work as a self-cleavage site in peptides [18], [33]. To our knowledge, there are no reports about the comparison of second gene expression dependent on adapter sequences. Probably, this F2A-dependant cleavage occurs more efficiently than internal ribosomal binding in mammalian cells. As Syzmczak et al. pointed out, the small 2A peptide at the end of the C-terminus of the protein may contribute to the antigenicity of proteins [32]. The F2A sequences used in this study were 2A sequences adjacent to a furin cleavage site; most of the Gag protein had no additional peptide at its C-terminus, and free-2A peptide should exist in mice [17]. However, in rare cases the Gag protein can still have a small peptide at its C-terminus. We investigated the immunogenicity of Gag, the first gene in the bicistronic vector, by the H-2K^d^/p24 tetramer assay. Significant differences in Gag-specific immunity were not detected between the three types of linkage (data not shown). There is not sufficient information regarding the safety of the F2A peptide in the clinical setting. Further research should be conducted for F2A-dependant bicistronic vectors with the aim of advancing towards clinical trials.

Although the efficiency of expression of the fusion protein was the second best in this study, the immunogenicity of Env induced by Ad-GE was approximately the same as Ad-G-IRES-E. Ad-GE expresses the Env protein not only as a single protein but also as a fusion protein (Fig. 6B). Whether processing of this fusion protein occurs in the proteasome or whether some TAP-dependent transaction steps take place in the antigen presentation cells has not been established. It is likely that the Gag part of the fusion protein was processed properly since immune responses to Gag were observed using the tetramer assay (data not shown). The accuracy of the processing of the Env part of the fusion protein was not clear. Further analysis should be conducted for the use of a fusion protein as an antigen.

Vaccination with recombinant Ad5 has achieved great success in inducing protection against virus infection in several animal models [34] and inducing HIV-specific responses in clinical trial [35]. Ad5 is well characterized, and its subclinical disease association in humans is well known [36]. However, a majority of the human population (more than 60%) is infected with the Ad5 virus [37]. The neutralizing antibody and the cellular immune responses against the Ad5 fiber and capsid may reduce the efficacy of the Ad5 vector when it is used in a clinical trial [38]. This study used a chimera Ad5 vector with Ad35 fiber, which relates with cell tropism. The Ad5/35, similar to Ad5, has a high productive titer in tissue culture cells, because it is commonly known that human subgroup B adenoviruses, such as Ad5, have a considerably higher titer as compared with other subgroup viruses, including Ad35. Nevertheless, the virus displayed the cell tropism of Ad35. Using the Ad5/35 vector, we found that high immunogenicity of the vaccine was observed in both mice and non-human primates [39], [40]. Coupled with the evidence that an Ad5/35 vector transduces human dendritic cells more efficiently as compared with an Ad5 vector [41], these findings suggest that the Ad5/35-HIV vector is a promising candidate for human trials.

In this study, the expression of the fusion type env protein in HeLa cells was higher than the IRES-dependant expression. A similar phenomenon has been reported, and the authors speculated that the efficiency of the reinitiation of translation of the second gene [42] could be dependent on the gene and cell combination. The precise mechanism leading to the higher efficiency of expression of the second gene in HeLa cells is unknown [16].

In summary, we have demonstrated that a CA promoter-controlled optimized HIV-1 clade C gene and an F2A-dependant second gene induced efficient gene expression and high antigen-specific immunity. These data should be taken into account for the use of these elements in the development of an HIV clade C vaccine and for gene therapy.

## Materials and Methods

### Cells

HEK293 and HeLa cells were cultured in Dulbecco's Modified Eagle's Medium (Wako Pure Chemical Industries, Ltd., Osaka, Japan) with 10% fetal calf serum.

### Plasmids

The native HIV-1 clade C gag gene (strain 96ZM651.8) was amplified from recombinant vaccinia virus DNA (vT331 obtained from AIDS Research and Reference Reagent Program, National Institutes of Health, Rockville, MD; Cat. No. 6523). The codon optimized HIV-1 clade C gag gene (gagopt) and envelope gp160 gene (envopt) were amplified from p96ZM651gagopt and p96ZM651gp160opt (obtained from the AIDS Research and Reference Reagent Program; Cat. No. 8675 and No. 8662), respectively. Three promoters were used to drive the expression of the antigen: the cytomegalovirus immediate-early 1 gene (CMV) promoter, the CMV promoter with the largest intron of CMV (intron A) sequence (CMVi), and the CMV enhancer and the chicken β-actin promoter with the chicken β-actin intron sequence (CA) (Fig. 1). Native gag (gag) or optimized gag (gagopt) were subcloned into the shuttle plasmid pHMCMV10 [12] under the control of the CMVi promoter to generate pCMVi-gag and pCMVi-gagpot, respectively. Optimized gag (gagopt) was subcloned in shuttle vector pHMCMV6 [43] or pHMCA5 [44] to generate pCMV-gagopt or pCA-gagopt, respectively. The shuttle vectors contain promoter-transgene-poly A cassette between the restriction enzyme sits, I-CeuI and PI-SceI.

To construct double gene expression plasmid, gagopt and envopt were cloned into the plasmid pIRES (Clontech, Mountain View, CA, USA) containing an IRES sequence. The gagopt-IRES-envopt fragment was then subcloned into the multiple cloning site of pHMCA5 [44], under the control of the CA promoter to generate pG-IRES-E. The gagopt, F2A and envopt genes were fused and subcloned into pHMCA5 to generate pG-F2A-E. The F2A oligonucleotides coding for the F2A peptide were synthesized based on the sequence RAKRAPVKQTLNFDLLKLAGDVESNPGP [32]. The fusion gene of gagopt and envopt was subcloned in pHMCA5 to generate pGE.

### Ad virus

I-CeuI/PI-SceI fragment of the shuttle plasmid containing the gene expression cassette was ligated into pAdHM34 [7], containing the E1/E3-deleted adenovirus type 5 genome with type 35 fiber. The resulting plasmid was digested with PacI and transfected into HEK293 cells with SuperFect Transfection Regent (Qiagen GmbH, Hilden, Germany) according to the manufacturer's instructions to generate the Ad virus. These Ad vectors were propagated in HEK293 cells and purified by CsCl_2_ gradient centrifugation; dialyzed with a solution containing 10 mM Tris (pH 7.5), 1 mM MgCl_2_, and 10% glycerol; and stored in aliquots at –80°C. Determination of the virus particle and infectious titers (plaque forming unit, PFU) was accomplished spectrophotometrically by the methods of Maizel et al. [45] and Kanegae et al. [46]_,_ respectively.

### Western blot

HeLa cells were transfected with plasmids using Lipofectamine 2000 Regent (Invitrogen, Carisbad, CA, USA) or infected with Ad vectors at a multiplicity of infection (MOI) of 5. After two days of incubation, the cell lysates were suspended with a low salt extraction buffer (10 mM Tris-HCl, 0.14 M NaCl, 1 mM dithiothreitol, 2 mM phenylmethanesulfonyl fluoride, 0.5% Nonidet P-40, and 3 mM MgCl_2_) for 30 min on ice. The lysates were mixed with an equal volume of 2 × sodium dodecyl sulfate (SDS) buffer (125 mM Tris-HCl, pH 6.8; 4% SDS; 20% glycerol; 0.01% bromophenol blue; and 10% beta-mercaptoethanol) and boiled for 10 min. The cell lysates were then loaded onto an 8% polyacrylamide gel. Proteins were transferred to a Hybond ECL nitrocellulose membrane (Amersham Pharmacia Biotech, Buckinghamshire, England). For primary antibodies, we used an HIV-1 clade C p24 mAb (lab prepared monoclonal antibody, unpublished) and an HIV-1 clade C gp120 mAb (laboratory-prepared monoclonal antibody, unpublished) to detect the Gag protein and the gp160 protein, respectively. Anti-β-Actin mAb (AC-15, Sigma, Saint Louis, MO, USA) was used as an internal control. A horseradish peroxidase (HRP)-conjugated anti-mouse IgG Ab (Cappel Laboratories Inc., PA, USA) was used as the secondary Ab. The blots were developed by using the ECL Plus western blotting detection system (Amersham Pharmacia Biotech).

### Quantitative Real-time PCR

HeLa cells were infected with Ad vectors at MOI of 2. After two days of incubation, total RNA was isolated from infected cells using ISOGEN (Nipongene, Toyama, Japan) and cDNA was generated using SuperScript™ II Reverse Transcriptase (Invitrogen, Carisbad, CA, USA). cDNA was used as a template for real-time PCR in triplicates with SYBR Premix Ex TaqTM II (TaKaRa, Shiga, Japan) and each gene-specific primers (gagopt; Forward: 5′-atcgaggaggagcagaacaa-3′, Reverse: 5′-gttctgcacgatggggtagt-3′, human Gapdh; Forward: 5′-ggtggtctcctctgacttcaaca-3′, Reverse: 5′-gtggtcgttgagggcaatg-3′). PCR and analysis were performed on an Applied Biosystems StepOne Plus Real-time PCR system (Applied Biosystems, Foster City, CA, USA). The expression of each gene was normalized with human Gapdh.

### Ad-mediated mouse immunization

Eight-week-old female BALB/c mice (H-2Dd) were purchased from Japan SLC Inc., Hamamatsu, Japan. The mice were intramuscularly administered with 1×10^8^ pfu of the Ad vector. The Ad vector carrying the CMV-LacZ-pA expression cassette was used as a negative control vector.

### Tetramer assay

The assay was performed as previously described [39], [47] A phycoerythrin (PE)-conjugated H-2K^d^/p24 tetramer (AMQMLKDTI) was prepared by the National Institute of Allergy and Infectious Disease MHC Tetramer Core Facility (Yerkes Regional Primate Research Center, Atlanta, GA, USA). In brief, 10 days post-immunization, peripheral blood mononuclear cells (PBMCs) or splenocytes were obtained from the immunized mice and stained with the tetramer and a fluorescein isothiocyanate (FITC)-conjugated anti-mouse CD8a Ab (Ly-2; eBioscience, San Diego, CA, USA) for 30 min at room temperature. The red blood cells were removed and the leucocytes were fixed with OptiLyse B Lysing Solution (Beckman Coulter, Fullerton, CA, USA) according to the manufacturer's instructions. Then the PBMCs or splenocytes were washed twice with the staining buffer (3% fetal calf serum (FCS), 0.09% NaN_3_ in phosphate-buffered saline (PBS)) and analyzed on a flow cytometer (Becton Dickinson, Franklin Lakes, NJ, USA) using CellQuest software (Becton Dickinson).

### Multicolor intracellular cytokine staining (ICS) assay

HIV-1 clade C Gag-specific, polyfunctional CD8^+^ T cells were detected 10 days after immunization with the Cytofix/CytoPerm Plus kit according to the manufacturer's instructions (Pharmingen, San Diego, CA, USA). In brief, PBMCs were obtained from immunized mice, and the red blood cells were removed by using a Lysing Buffer (BD Bioscience Pharmingen, Franklin Lakes, NJ, USA). Lymphocytes were incubated with 10 µg/ml of HIV-1 clade C p24 peptide (AMQMLKDTI) and a FITC-conjugated anti-mouse CD107a Ab (1D4B; Southern Biotechnology Associates Inc., Birmingham, AL, USA) for the detection of Gag-specific responses in a 24-well plate for 1 h at 37°C, followed by an additional 5 h in the presence of 1 µg/ml of BD GolgiStop (BD Bioscience Pharmingen). The cells were washed with staining buffer and stained with allophycocyanin (APC)-Cy7-conjugated anti-mouse CD8a Ab (53-6.7; BioLegend, San Diego, CA, USA) at 4°C for 30 min. The cells were suspended in 250 µl of Cytofix/Cytoperm solution at 4°C for 15 min, washed with Perm/Wash solution (BD Bioscience Pharmingen), and stained with PE-conjugated anti-mouse IFN-γ Ab (XMG1.2, eBioscience) and APC-conjugated anti-mouse TNF-α Ab (MP6-XT22, eBioscience) for 30 min at 4°C. The cells were acquired by Moflo cytometer (Beckman Coulter, Fullerton, CA, USA). Data were analyzed using FlowJo (Treestar).

To detect env-specific cellular immunity responses, we used a 15-mer amino acid peptide pool with 11-mer overlap of HIV consensus subtype C env (obtained from AIDS Research and Reference Reagent Program; Cat. No. 9499). The peptide pool contains 20 peptides ranged from No. 721-811 amino acid of HIV clade C env (peptide Cat. No. 9365-9384). Splenocytes were used for detection of env-specific cytokine secretion using ICS assay as detection of gag-specific immunity responses (2 µg/ml per peptide was used for stimulation).

### Enzyme-linked immunosorbent assay

The HIV-1 clade C p24 protein [48] or envelope V3 peptide (NNTRQSIRIGPGQTFYATGDIIGD) were used for the detection of HIV-1 clade C Gag-specific Ab or HIV-1 clade C gp160 Ab, respectively.Briefly, 96-well microtiter plates were coated with 10 µg/ml of HIV clade C p24 peptide or V3 peptide and incubated overnight at 4°C. The wells were blocked with PBS containing 1% BSA for 2 h at 37°C. They were then treated with 100 µl of diluted sera and incubated for an additional 2 h at 37°C. The bound immunoglobulin was quantified using an HRP-conjugated anti-mouse IgG Ab (diluted 1∶1,000; Cappel, Laboratories Inc., PA, USA). The mean Ab titer was measured at a wavelength of 450 nm by using a microplate reader (Model 450; Bio-Rad, Hercules, CA, USA).

### Data analysis

All results were expressed as the mean ± standard error of the mean. Statistical analysis of the experimental and control data was performed using a one-way factorical analysis of variance. *P*-value<0.05 defined statistically significant.
